# Complications after cosmetic periocular filler: prevention and management

**DOI:** 10.20517/2347-9264.2020.133

**Published:** 2020-08-15

**Authors:** Mike Zein, Ryan Tie-Shue, Nathan Pirakitikulr, Wendy W. Lee

**Affiliations:** 1 Mcknight Vision Research Center, Bascom Palmer Eye Institute, University of Miami-Miller School of Medicine, Miami, FL 33136, USA.; 2 Department of Biomedical Research, Yale University, New Haven, CT 06520, USA.; 3 Division of Oculofacial Plastic and Reconstructive Surgery, Department of Ophthalmology, Bascom Palmer Eye Institute, University of Miami-Miller School of Medicine, Miami, FL 33136, USA.

**Keywords:** Fillers, hyaluronic acid, rejuvenation, periorbital, aesthetic, skin necrosis, complications, blindness

## Abstract

Soft tissue fillers are a mainstay in contemporary, minimally invasive facial rejuvenation procedures owing to timely results and minimal recovery period. Although associated with a low complication rate, soft tissue fillers are not without risk. Complications range from mild superficial skin irregularities to granuloma formation to vascular occlusion leading to skin necrosis or even blindness. Fillers vary in composition, elasticity, hydrophilicity and duration of effect that is tailored to specific cosmetic indications. Selecting the right product for the desired effect can cut down on unwanted outcomes. Severe adverse events can be avoided with safe injection technique, early recognition of symptoms and a thorough knowledge of the local anatomy. This review outlines several complications all providers should recognize and discusses strategies for their prevention and management.

## INTRODUCTION

Soft tissue fillers have become an increasingly popular intervention for facial rejuvenation over the past two decades with quick results, minimal recovery time and relatively low complication rate. Since their approval by the FDA in 2004, the number of hyaluronic acid (HA) filler injections performed in the United States has steadily risen to nearly 2.7 million procedures per year as of 2018^[1]^. As a naturally occurring component of skin and connective tissue, HA is highly biocompatible and non-immunogenic. HA is a favored choice for patients with little to no history with injectables as its effects are temporary, lasting between 6 to 24 months with natural degradation. This process can be accelerated with the use of hyaluronidase providing some ability to reverse unwanted effects.

While soft tissue fillers and HA in particular are non-incisional and less invasive than other interventions for facial rejuvenation, they still carry a number of risks when performed without proper precautions. In the United States, soft tissue filler injections are performed by a wide variety of health care providers, including but not limited to facial plastic surgeons, dermatologists, oculoplastic surgeons, plastic surgeons and the nurse practitioners and physician assistants working under their supervision. While such providers may be fully licensed to perform these procedures, there are considerable differences in training, familiarity with relevant anatomy, and ability to manage complications. Furthermore, the black market in cosmetic fillers and ready availability of unlicensed injectors provides a steady source of complications that licensed providers should be prepared to encounter^[2,3]^. Between 2013 and 2017 over 2800 reported adverse events occurred in the United States according to FDA databases^[4]^.

Complications vary and range from the mild, self-resolving ecchymoses to the more persistent irregular surface contours, festoons, and the bluish hue (Tyndall effect) seen with superficial filler placement. Severe granulomas have also been seen long after filler injection. The most severe complications are due to filler vascular occlusion, which can result in skin necrosis and sometimes irreversible vision loss^[5]^. This review focuses on filler-associated complications that are most commonly encountered by ophthalmologists and oculoplastic surgeons, and addresses various preventative and management strategies.

## AVOIDING VASCULAR COMPLICATIONS

A thorough knowledge of the local anatomy, use of safe injection techniques and timely recognition of symptoms can help minimize the risk of the most severe complications. The face and periorbita are supplied by a rich network of blood vessels that communicate through complex anastomoses. Iatrogenic perforation or cannulation of the arterial wall during filler injection can introduce emboli that may cause vaso-occlusion either up- or downstream of the site of injection^[6]^.

### Pertinent anatomy

**Figure 1.**
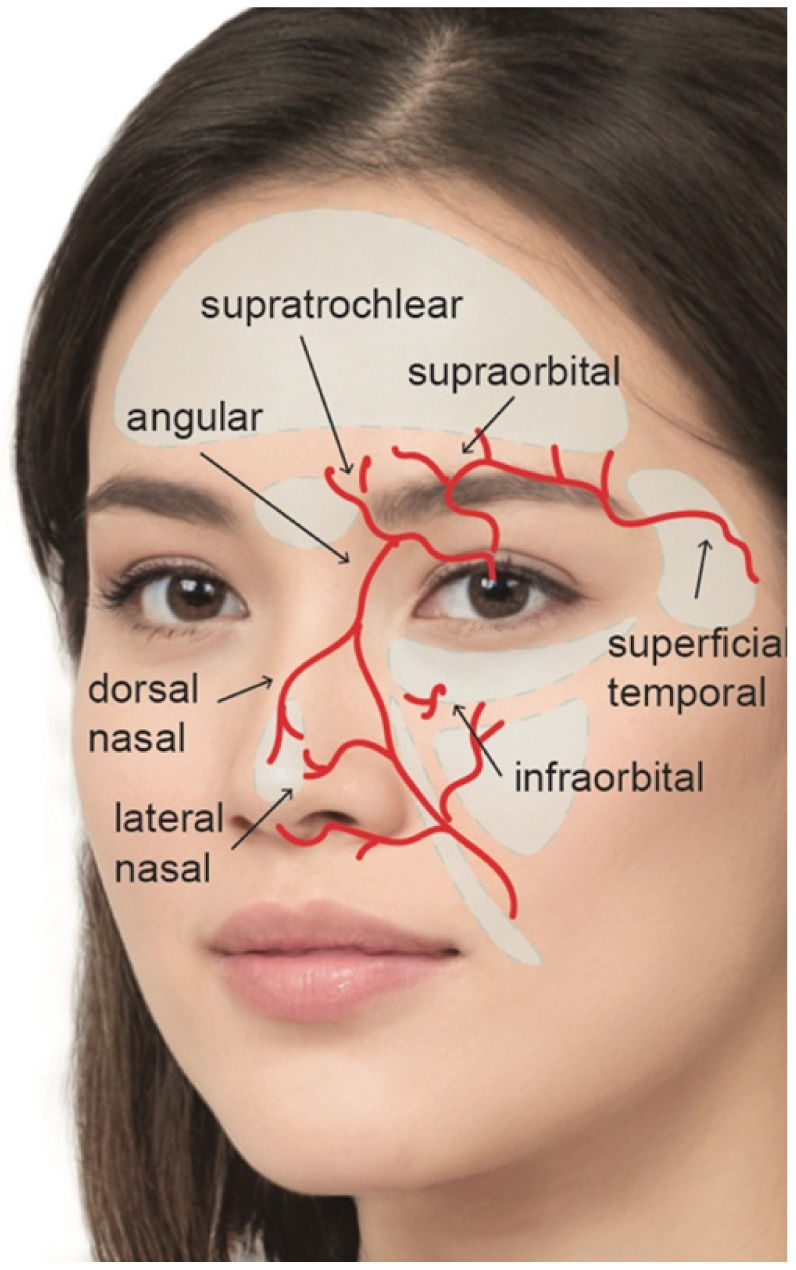
Vascular anatomy relevant to cosmetic filler injection and their relation to commonly targeted zones including the forehead, glabella, temporalis fossa, tear trough, midface, nasolabial groove and nasal dorsum

Particularly high-risk zones for vascular complications include the glabella, temporalis fossa, tear trough, midface, nasolabial grooves, and nasal dorsum owing to the large vessels in these areas [Figure 1]^[7]^. In the glabellar region, the supratrochlear branch of the ophthalmic artery exits along the orbital rim approximately 2 cm lateral to midline superficial to the corrugator and deep to the orbicularis and frontalis, before becoming more superficial and entering the subcutaneous plane 2 cm above the orbital rim. The supraorbital branch of the ophthalmic artery exits along the orbital rim through the supraorbital notch approximately 3 cm lateral to midline. Similar to the supratrochlear artery, the supraorbital artery courses deep to the orbicularis and frontalis before entering the subcutaneous plane anywhere from 2 to 6 cm above the orbital rim^[8,9]^.

The temporalis fossa consists of skin, subcutaneous fat, temporoparietal fascia, superficial and deep temporal fascia surrounding loose areolar tissue, temporalis and periosteum. The frontal branch of the superficial temporal artery courses within the temporoparietal fascia approximately 2 cm superior and lateral to the peak of the brow. The middle temporal vein and the temporal branch of the facial nerve also course near this region, posing the additional risk of pulmonary embolism and nerve injury^[10]^. There is also a connection between the temporal fossa and the orbit. The zygomatico-temporal artery connects the anterior deep temporal artery to the lacrimal artery and runs through the zygomatico-temporal foramen. This foramen is located on the posterior surface of the zygomatic bone. Filler injected in the region of this foramen could potentially travel directly into the orbit via this route.

In the tear trough and midface regions, the infraorbital artery and nerve emerge through the infraorbital foramen approximately 3 cm lateral to midline just inferior to the orbital rim^[11,12]^. Along the nasolabial groove, the facial artery and its branches course in a highly variable pattern. The facial artery can be found medial, lateral or crossing the nasolabial folds. On average it is found 1.7 mm medial to the folds at the upper middle third and 0.3 mm medial at the lower middle third^[13]^. The inferior alar artery and lateral nasal artery branch off from the facial artery at the level of the ala. At the takeoff of the lateral nasal artery, the facial artery becomes more superficial and continues as the angular artery, which crosses the nasojugal groove medially where it is prone to injury during tear trough injections.

After branching into the inferior alar branch and lateral nasal artery, the angular artery continues towards the medial canthus and connects to the dorsal nasal arterial system. The nasal dorsum contains a larger arterial and venous system superficial to nasal musculature in the subcutaneous plane. Sparse vascular networks are located within the areolar layer, including a marginal artery, which courses over the lower lateral cartilage caudal border, and the dorsal nasal artery, a terminal branch of the ophthalmic artery that courses above the muscular layer. Both of these arteries course superficially towards the nasal tip. Each of the arteries listed above have a connection with the ophthalmic and central retinal arteries so injection in each of these areas carries the risk of blindness.

### Principles of safe injection

A safe injection begins with selecting the appropriate needle. We advocate the use of smaller needles such as 30 G for injecting into the superficial and mid-dermis, and 27 G for deeper injections. Some injectors recommend the use of large (25 or 22 G) blunt cannulas as they may provide a lower risk of iatrogenic vessel puncture. After the patient has been properly cleansed and anesthetized, digital pressure can be used to mark and occlude vessels at anatomic landmarks to prevent inadvertent puncture and back flow^[14,15]^. Needles should be introduced perpendicular or parallel to vessels with the tip pointed towards the direction of arterial flow. If a cannula is being used, it should always be parallel to the vessel. Prior to injecting, the syringe is gently aspirated to ensure no blood return, though the absence does not guarantee a vessel has not been punctured^[16]^. The product is then introduced at low flow to avoid overcoming mean arterial pressure should intra-arterial cannulation occur. Keeping the needle in constant motion during injection will also help prevent product from depositing inside a vessel. If resistance is felt, then the needle should be repositioned.

As product is injected, the needle tip should be maintained at a depth to minimize contact with any vessels. The appropriate depth depends on the region being treated. In the glabella the needle tip is kept either superficially in the dermis or deep to the frontalis muscle to avoid vessels in the subcutaneous plane. In the temporalis fossa, filler is placed under the temporalis muscle to avoid vessels coursing in the temporoparietal fascia^[17]^. As an alternative, a cannula can be used in more superficial tissues of the temple. In the tear trough and midface, filler should be placed just above the periosteum with special care medially where the infraorbital canal is located. If filler is needed medially, it can be injected laterally then massaged into place.

Safely injecting along the nasolabial groove can be challenging due to the variable course of the facial artery. In the lower two-thirds, the facial artery generally runs at the muscular plane or deeper. We therefore advocate keeping the needle tip in the superficial subcutaneous plane in this region and then moving deeper to the preperiosteal plane near and above the alar base. At the nasal dorsum the vascular network is located entirely in the subcutaneous plane so injections are kept deep in the preperichondrial and preperiosteal planes.

Vascular compromise must be treated urgently once recognized. All filler procedures must be performed with a “filler crash cart” in the immediate vicinity. This kit should contain at minimum 10 vials of unexpired hyaluronidase (e.g., Hylenex, Halozyme Therapeutics, San Diego, CA; Vitrase, Bausch & Lomb, Irvine, CA). Other items recommended by many providers include aspirin to prevent clot propagation, and warm compresses and nitroglycerin to promote vasodilation. A quick referral system for ophthalmic care should be in place in the event the ophthalmic artery is injured.

### Signs and symptoms of vascular injury

**Figure 2.**
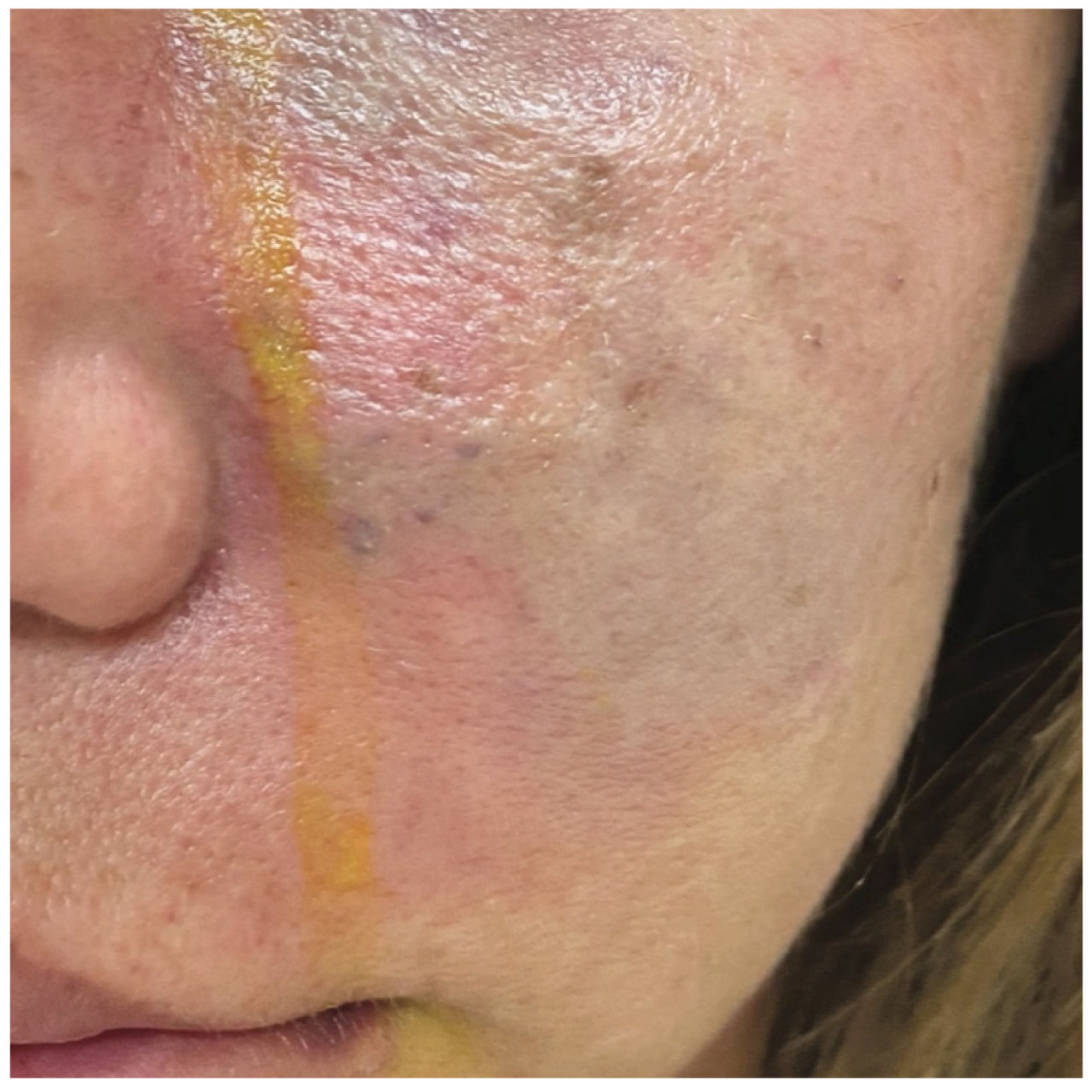
A 38-year old female developed pain, redness and swelling of the left cheek hours after Radiesse injection to the zygoma for midface augmentation. She came to our care two days later. Note the erythema and dusky appearance to the skin in the distribution of the infraorbital artery and facial artery. The patient developed pustules and sloughing the following day. She was treated with aspirin, topical nitroglycerin, oral prednisone, intradermal sodium thiosulfate, hyperbaric oxygen and manual debridement with gradual improvement in tissue perfusion over two weeks and minimal scar tissue buildup

Vascular injury can cause a range of complications. Early signs that an artery has been punctured include the appearance of blood upon aspiration, formation of a hematoma, skin blanching or discoloration, and intense pain at the injection site^[18]^. Should any of these occur, the procedure must be aborted immediately. If not promptly recognized, continued injection after intra-arterial cannulation can lead to vascular occlusion resulting in skin necrosis or worse, vision loss. Skin necrosis occurs in < 0.001%-0.5% of patients but accounts for 43% of serious complications related to soft tissue filler injections in the MAUDE database^[19]^. Early on the skin takes on a blanched then mottled and dusky appearance in the distribution of the injured vessel [Figure 2]. The surrounding area may also appear erythematous.

Emboli can also travel retrograde up through the ophthalmic artery resulting in ocular complications^[20]^. The first reported case of vision loss due to cosmetic soft tissue filler injection occurred in 1988 and was due to retinal artery occlusion^[21]^. These patients report sudden profound unilateral vision loss, but may have preservation of central vision if the cilioretinal artery is spared^[22]^. Emboli traveling to other branches of the ophthalmic artery can result in ischemic optic neuropathy presenting with altitudinal vision loss^[23,24]^ or oculomotor nerve palsy presenting with diplopia and in some cases ptosis^[25-27]^. Complete occlusion of the ophthalmic artery results in orbital infarction syndrome which is characterized by severe orbital pain, vision loss, loss of extraocular motility and ptosis. On ophthalmic exam these patients also demonstrate hypotony, corneal edema and persistent ocular inflammation owing to poor perfusion of all ocular structures^[28-30]^. The number of ophthalmic complications related to cosmetic filler continues to rise with the growing demand for these procedures. In the period between January 2015 and September 2018, there were 48 published cases of filler induced ophthalmic complications; the majority were related to vision loss^[31]^.

Certain fillers are associated with a greater risk of vascular compromise owing to differences in particle size, viscosity, cohesivity and effect on inflammation and clotting [Table 1]^[16,32-35]^. The incidence of vascular occlusion from hyaluronic acid fillers has been reported to be approximately one tenth the overall incidence from all fillers, which includes polymethyl-methacrylate microspheres (Bellafill, Suneva Medical, San Diego, CA), calcium hydroxylapatite (e.g., Radiesse, Merz Pharmaceuticals, Greensboro, North Carolina) and poly-L-lactic acid (e.g., Sculptra, Valeant Aesthetics, Bridgewater, New Jersey)^[36]^. The risk is also highest from injections of the glabellar and nasolabial regions^[19,37]^. If a hyaluronic acid product was injected, the area should be immediately flooded with injections of hyaluronidase. While allergic reactions to hyaluronidase have been reported, in the acute setting the benefits of this therapy far outweigh the risks^[38]^. For other products topical nitroglycerin can be considered and warm compresses can be rapidly applied, and if available the patient can be referred for hyperbaric oxygen. Few reports suggest sodium thiosulfate can be used to dissolve calcium hydroxylapatite^[39,40]^. However, apart from injection of hyaluronidase, no other therapy has been proven effective^[41]^. There is no consensus on how to treat vision loss from filler-associated vascular occlusion, though anterior chamber paracentesis, ocular massage, hyperbaric oxygen and retrobulbar hyaluronidase injection have all been tried^[37,42]^. Fortunately, with proper attention to anatomy vascular complications are exceedingly rare.

## AVOIDING BAD COSMETIC OUTCOMES

### Strategies for filler placement

There are four basic injection techniques associated with dermal fillers. The most basic technique is threading, which involves the application of a continuous line of filler injected in a retrograde fashion to correct discrete rhytids. The crosshatching technique builds upon this and involves continuous overlapping horizontal and vertical lines to build volume. The third technique is fanning, which involves drawing filler lines in a fan-shaped projection. Finally, serial puncture involves the injection of discreet aliquots of product to correct deep deformities.

### Irregular surface contours

Superficial irregularities are commonly encountered in regions with minimal subcutaneous fat. To avoid this complication, filler must be placed deeply. In the tear trough region, we use a serial puncture technique to advance the needle to the periosteum along the inferior orbital rim and deliver small boluses of product working from medial to lateral. Similarly, in the temporalis fossa, we inject deeply under the fascia of the temporalis muscle in the area of maximal volume loss attempting to avoid vessels and nerves while providing a nice contour. In areas that require more superficial injection such as along the nasolabial groove, using hyaluronic acid fillers with higher cohesivity will diminish surface irregularities^[43]^. Any lumps or bumps noticed early on are addressed with manual massage, while persistent irregularities generally must be dissolved with hyaluronidase.

### Festoons

Festoons or chronic fluid collections often become more noticeable following filler injection with particular hydrophilic hyaluronic acids. Tear trough filler is particularly prone to this complication and it is therefore important to select a product with low water affinity such as Restylane-L (Galderma, Lausanne, Switzerland).

### Tyndall effect

The Tyndall effect is characterized by a blue-grey discoloration of filler placed superficially under the skin. This occurs primarily in the tear trough region where there is minimal subcutaneous fat and the overlying skin is very thin. The phenomenon is due to the scattering of blue light as it passes through small particles in suspension^[44,45]^. As all fillers are similarly prone to this complication, the primary preventative measure is deep injection. Belotero Balance (Merz, Frankfurt, Germany) is made with varying particle sizes, a product characteristic thought to decrease the chances of the Tyndall effect.

## OTHER COMPLICATIONS

### Granulomas

Granulomas may appear anywhere from 6 to 24 months after injection, and is estimated to occur in 0.1%-1% of patients^[46]^. Presentation usually consists of a constellation of swelling, tenderness, erythema and possible suppuration^[19,47]^. The foreign body reaction results in prolonged inflammation that results in the formation of a nodule comprised of macrophages producing inflammatory products^[48]^. HA fillers reinforced with hydroxyethyl-methacrylate fragments have been associated with late-onset granuloma formation^[49]^. It is postulated that impurities and surface irregularities associated with formulations containing particles < 20 μm in size, are phagocytosed which propagates granuloma formation^[47,50-52]^. Granulomas typically resolve within two years without the need for intervention, but if the lesion persists can be treated with intralesional corticosteroids or surgical excision. Supplemental laser resurfacing or dermabrasion can help improve superficial surface irregularities^[53]^. Attempt at dissolving the granulomas with hyaluronidase is also a good option.

### Infections

Infections, while rare, can present in a variety of forms, ranging from erythematous nodules to abscesses. The most common culprits are usually bacterial skin flora (*Streptococcus pyogenes* and *Staphylococcus aureus*), or in some cases herpes simplex virus^[18,48,54]^. In some cases, latent infections or atypical bacteria can be explained by biofilm formation^[55]^. In the event of an abscess, incision and drainage is indicated followed by empiric antibiotics to cover the previously mentioned bacteria. Prophylaxis for herpes simplex infections is indicated for patients with a history of cold sore outbreaks.

## CONCLUSION

With the growing number of filler injections used, awareness and prevention of ocular and facial complications is paramount. While the majority are self-resolving, such as ecchymoses at the injection site, ocular complications such as irreversible blindness and orbital and ocular ischemia are detrimental to patients’ visual prognosis and general quality of life. Through a strong grasp of the local anatomy, periodicity and presentation of complications, safe technique and knowledge of the available formulations on the market, these adverse events can be mitigated or prevented completely.

## Figures and Tables

**Table 1.** Summary of hyaluronic acid dermal fillers currently commercially available in the United States

Product	Site	[HA] (mg/mL)	G'(Pa)	Duration (months)
Belotero Balance	Superficial - mid-dermis	22.5	30	6
Restylane-L	Superficial - mid-dermis	20	565	6
Restylane	Medium - deep	20	544	9
Restylane Silk	Superficial - sub-mucosal	20	344	6
Restylane Lyft	Medium - deep	20	545	9
Restylane Refyne	Medium - deep	20	47	12
Restylane Defyne	Medium - deep	20	260	12
Restylane Kysse	Superficial- sub-mucosal	20	156	12
Juvéderm Ultra XC	Superficial - medium	24	207	12
Juvéderm Volbella XC	Superficial - medium	15	274	12
Juvéderm Vollure XC	Medium - deep	17.5	317	18
Juvéderm Voluma XC	Medium - deep	20	353	24
Juvéderm Ultra Plus XC	Medium - deep	24	244	12
Revanesse Versa Plus	Medium - deep	28	130	12
Teosyal RHA 2	Superficial - mid-dermis	23	144	15
Teosyal RHA 3	Medium - deep	23	184	15
Teosyal RHA 4	Deep - sub-cutaneous	23	296	15

[HA]: concentration of hyaluronic acid, G’: elastic modulus. Values were obtained from manufacturer documentation and published sources^[56-60]^. Exact G’ values varied between sources, but the relative differences between listed products were similar
